# Interoceptive Processes in Anorexia Nervosa in the Time Course of Cognitive-Behavioral Therapy: A Pilot Study

**DOI:** 10.3389/fpsyt.2016.00199

**Published:** 2016-12-15

**Authors:** Dana Fischer, Götz Berberich, Michael Zaudig, Till Krauseneck, Sarah Weiss, Olga Pollatos

**Affiliations:** ^1^Clinical and Health Psychology, Institute of Psychology and Education, Ulm University, Ulm, Germany; ^2^Klinikum Windach a. Ammersee, Windach, Germany; ^3^kbo-Isar-Amper-Klinikum, Munich-Haar, Germany; ^4^AMC – Wolfart Klinik, Zentrum für Adipositas- und Metabolische Chirurgie, Munich-Gräfelfing, Germany

**Keywords:** anorexia nervosa, interoceptive accuracy, interoceptive sensibility, eating disorders, heartbeat perception, cognitive-behavioral therapy

## Abstract

**Objective:**

Previous studies report reduced interoceptive abilities in anorexia nervosa (AN) using various methods. Recent research suggests that different levels of interoceptive processes aiming at different subdomains of interoceptive abilities must be further distinguished as these levels can be differentially affected. Two important levels refer to interoceptive accuracy (IA) derived from objective performance tasks such as the heartbeat detection task and interoceptive sensibility (IS) as assessed by self-report. There is a lack of studies investigating both IA and IS in AN and examining them in the time course of therapy. The aim of this pilot study was to evaluate the different interoceptive processes – especially IA and IS – in the time course of therapy.

**Methods:**

Fifteen patients with AN (restricting type) from the Psychosomatic Clinic in Windach were investigated three times (T1, T2, T3) during a standardized cognitive-behavioral therapy and compared with 15 matched healthy controls assessed at Ulm University in a comparable design. All participants performed the heartbeat detection task examining IA and completed standard psychological assessments including an assessment of IS.

**Results:**

Patients with AN showed a significantly decreased weight, higher levels of depression, and both reduced IA and IS compared to healthy controls at T1. Following therapy, patients recovered in terms of weight and depression symptomatology. A descriptive trend for recovering from IA and IS was observed.

**Discussion:**

Our findings suggest that interoceptive deficits are present in recovered patients. Therefore, further investigations are needed with more patients, differentiating between relapsed and recovered patients, and more specific training methods to improve interoceptive processes.

## Introduction

Anorexia nervosa (AN) is a psychiatric disorder with an excessive weight loss (1). Patients are characterized by fear of gaining weight, rejection of their body, and a disturbed body image (1–3). Patients restrict their food intake, especially food with a high-fat content and follow an excessive exercise program (1, 4). For this type of eating disorder, severe psychological, physiological, and social consequences are observed. This includes for instance declining interests for other people, comorbidities like depression or anxiety disorder, impaired concentration, loss of sexual interests, and amenorrhea. Previous studies suggest that patients with eating disorders (ED) and especially patients with AN have difficulties in the perception of bodily signals (interoception), mostly assessing interoceptive deficits by self-report or questionnaire [interoceptive sensibility (IS); see Ref. (5–8)].

Interoception is understood as the body-to-brain axis of sensation concerning the state of the internal body and its visceral organs (9, 10). The general concept of interoception includes two forms of perception: proprioception (signals from the skin and musculoskeletal apparatus) and visceroception [signals from the inner organs like heart rate, breath, and hunger (9–13)]. Interoceptive accuracy (IA) is one way of quantifying interoceptive processes. This variable describes the ability to perceive or detect bodily internal signals [e.g., heartbeat, respiratory, or gastric signals (9, 12, 14)]. Many studies found interindividual differences regarding the perception of cardiovascular (15–18) and gastrointestinal signals (19), with an observed significant overlap between accurate detection of heartbeats and gastrointestinal signals (20, 21), suggesting a general accuracy for visceral processes across different modalities.

The individual differences to perceive internal bodily signals can be characterized as a trait-like sensitivity toward one’s visceral signals, which are understood as long-term results of “visceral” learning processes depending on autonomic reactivity during different situations of daily life that evoke substantial changes in autonomic activity (13, 22). Several studies used different methods to manipulate autonomic cardiovascular activity and to achieve a modulation of them (13, 21, 23–25). Recent findings stem from research in which tasks were used that provoked an increased focus on the self-aspect and enhanced aspect of self-processing (26). A similar effect was observed when participants gazed at a photograph of their own face or at self-relevant words (27).

Garfinkel and Critchley (28) highlighted the necessity to distinguish between different levels of interoceptive processes, suggesting the term IA when referring to behavioral testing such as performance on heartbeat perception tests, IS when referring to subjective beliefs about the individual perception of bodily internal signals like hunger and satiety, and metacognitive awareness when assessing confidence-accuracy correspondence. Referring to existing literature and having these definitions in mind, the question whether interoceptive processes are abnormal in AN becomes more complex. Although disturbances in IS as assessed by questionnaire are very common in AN [e.g., Ref. (6, 7)], deficits in IA are found less consistently (5, 29). Concerning interoceptive processes in ED, a recent study by Pollatos and Georgiou (30) could demonstrate that bulimic patients show an inverse correlation between different levels of interoceptive processes as measured by IA and IS, which is not the case for healthy controls. A former study in anorectic patients could also demonstrate that both IA and IS were reduced in anorectic patients, yet interestingly both aspects were not correlated (5). This could imply that IA and IS might describe rather different and presumably independent levels of perceiving the own bodily signals. Mehling et al. (31) emphasize that subjective beliefs are related with cognitive processes such as attention, appraisal, belief, memories, and attitudes (31) as well as usually cover a large variety of internal bodily signals, while typical tasks measuring IA target presumably the heartbeat, or other single internal systems such as respiratory or gastric signals (32). These results suggest that several levels of interoceptive signal processing might be affected differentially in AN.

In a recent study, McFadden et al. (33) reported alterations in brain activation patterns contrasting anorectic females and recovered anorectics. They reported that the alterations in the default network observed only in women with AN suggest state-dependent abnormalities that could be related to altered interoception and body image in these women when they are underweight but that remit following recovery. These results suggest that certain aspects of interoceptive processes may be altered during therapy, while others remain affected. The question whether different aspects of interoception including IA and IS change during the time course of therapy and whether this effect is driven by an increase in body weight has, to our knowledge, not been addressed yet and should be elucidated in this study. Although there is evidence for improvement of IS following therapy (6), research on possible improvement of IA in AN during therapy is still lacking. Therefore, the current study investigated anorectic patients from the Psychosomatic Clinic Windach am Ammersee during the time course of stationary therapy and compared them with matched healthy controls. We hypothesized that patients with AN show an increase of IA and IS over time by a cognitive-behavioral therapy in comparison to healthy controls.

## Materials and Methods

### Participants

Fifteen female patients who met the criteria for AN as defined by the International Classification of Disease 10 (ICD-10) criteria were recruited from the Psychosomatic Clinic Windach am Ammersee, Germany. The patients’ diagnoses were determined according to ICD-10 criteria based on semi-structured clinical interviews administered by a senior staff member. All participants received a cognitive-behavioral therapy with special attention to maladaptive emotional processes and the systemic context. They took part in a group therapy and additional individual therapy sessions. Aim of the in-patient intervention is a normalization of the eating behavior and an adequate body weight [minimum body mass index (BMI) of 19]. In addition, therapists target etiological factors for development and maintenance of AN. The therapeutic concept includes aspects of confrontation and aim at interrupting as well as alternative handling of the problematic eating behavior. The three predetermined meals per day were administered together with all members of the group therapy. At the beginning of therapy, participants had to stop all intake of aperients or diuretics. The patients agreed with the therapists on an individual target weight and a weight gain of 700 g/week. If the patients fail this goal more than twice, they have to take a break from the therapy with the opportunity of resumption. Adjuvant other therapies like art therapy, body therapy, or nutrition counseling are part of the treatment concept.

Interoceptive accuracy, IS, and possible confounding variables such as BMI and depression score were assessed for three times based on the therapy process at the beginning (T1), after 4–6 weeks following an increase of two BMI points (T2), and at the end of therapy (T3). Other experimental tasks and measurements also taken are not reported here. Patients with AN started with the study in the first or second week of their therapy. The last assessment was after 12–14 weeks, based on the average duration of the patients stay. Patients with AN had an mean age of 27.4 years (SD = 7.8) and mean BMI of 15.7 (SD = 1.3, see Table 1) at T1. Furthermore, patients reported their educational level choosing between (1) without any educational qualification (*n* = 1); (2) secondary general school certificate (*n* = 1); (3) intermediate school certificate (*n* = 5); (4) entrance qualification for technical college (*n* = 5); and (5) entrance qualification for university (*n* = 3). Concerning former therapy experience, four patients had once and one patient had twice therapy in an ambulant setting, additionally four patients were once, five patients twice, and one patient more than three times in a hospital treatment before.

**Table 1:** **Descriptive variables of AN patients and controls at T1**.

	AN	Controls	*t* (df = 28)	*p*
	Mean (SD)	Mean (SD)		
Age (years)	27.4 (7.8)	27.9 (7.6)	−0.19	0.851
Education	3.13 (1.0)	3.4 (0.9)	−1.01	0.319
BMI	15.7 (1.3)	21.0 (1.8)	−9.17	<0.001
IA	0.53 (0.1)	0.70 (0.2)	−3.73	0.002
IS (EDI-2)	35.47 (6.7)	21.60 (4.5)	6.65	<0.001
BDI-II	25.0 (11.0)	4.6 (1.7)	7.09	<0.001

*AN, anorexia nervosa; BMI, body mass index; IA, interoceptive accuracy; IS, interoceptive sensibility; BDI-II, Beck Depression Inventory-II*.

Concerning comorbidities, four patients had a depression and were treated with antidepressants (SSRIs), and one patient had a personality disorder (borderline). Referring to the Beck Depression Inventory (BDI) scores, additional seven patients expressed increased depressive symptoms without fulfilling the criteria for a depressive episode (BDI > 20). Three patients were treated because of the following somatic disorders: one patient because of hypothyroidism, one because of tension headache, and one because of gastritis. In addition, six patients took some dietary supplements (e.g., iron-rich pills). Finally, patients with any purging at the moment or former diagnosis of bulimia nervosa were excluded, resulting in patients with a restricting type of AN only.

The control sample consisted of 15 healthy females recruited by staff or students at Ulm University. These healthy controls were matched according to age and educational level. At the end of the study, they received a compensation of € 20. In the control group, the mean age was 27.9 (SD = 7.6), mean BMI was 21.0 (SD = 1.8), and mean educational level was 3.4 (SD = 0.9) at T1. Regarding the BDI value, none of the participants showed a pathological value (BDI > 8). Exclusion criteria assessed by an anamnestic questionnaire were any kind of medication intake (except of contraceptives), past or current disorders regarding eating disorder, other psychiatric as well as somatic disorders.

### Ethics Statement

The study was carried out in accordance with the Declaration of Helsinki; ethical approval was obtained from the Institutional Review Board of the Ulm University. Before testing, informed consent was obtained.

### Instruments

An anamnestic questionnaire explored health status and personal data (e.g., age, educational background). All participants received a questionnaire battery with standard psychological questionnaires.

Interoceptive accuracy was assessed by the Mental Tracking Method by Schandry (34). The task started with a short training interval of 10 s, which was followed by three intervals of 25, 45, and 35 s. During these intervals, participants should focus on their heartbeat, count them silently, and report the counted heartbeats verbally. They had no information about the length of these intervals, and the supervisor gave the start/stop signal for each interval. Furthermore, participants were instructed to sit relaxed and not to talk as well as not to evaluate their pulse or use other manipulating strategies to support the perception of their heartbeats. For the objective measurement of heartbeats, the POLAR RS800CX heart rate monitor (Polar Electro Oy, Kempele, Finland, sampling rate of 1000 Hz) was used. The advantage of this sports watch is the easy and non-invasive use as well as the valid and reliable recording compared to other ECG measurement devices (35–37). The corresponding Polar Pro Trainer 5 Software (version 5.40.172) was used to perform analyses of the cardiovascular signals. Finally, a mean heartbeat perception score or interoception score was calculated, which includes a ratio between the objective/recorded and the subjective/counted heartbeats. Therefore, the following transformation was approved:

$$\frac{1}{3}\sum \left(1-\frac{\left(\left|\text{recorded heartbeats}-\text{counted heartbeats}\right|\right)}{\text{recorded heartbeats}}\right)$$

Interoceptive accuracy scores range from 0 to 1, with higher scores indicating small differences between the counted and recorded heartbeats and consequently a better IA. Several studies reported mean heartbeat detection scores varying between 0.65 and 0.70, e.g., Kever et al. (38) reported a mean score of 0.69 (SD = 0.18, range = 0.16–1.00) in more than 400 healthy participants [see also Ref. (39–41)]. The heartbeat perception method is well validated and has a good test–retest reliability (34, 42, 43).

For investigation of IS, we administered the subscale “interoceptive awareness” from the Eating Disorder Inventory-2 [EDI-2; (44, 45)]. This subscale comprises the subjective identification of bodily signals, especially hunger and satiety. Questions are rated on a six-point scale, ranging from 1 (never) to 6 (always). High scores indicate problems in IS. The median value for female healthy participants is 22.0 for the subscale of “interoceptive awareness.” In comparison to healthy controls, patients with AN (restricting type) show a median value of 32.0 referring to Paul and Thiel (45).

The BDI-II quantifies depressive symptoms in the last week on a four-point rating scale [0 = not at all, 3 = always; (46)]. This widely used and well-validated 21-item self-report inventory of depressive symptomatology provides scores ranging from 0 to 63. The Cronbach’s α for the questionnaire differs between 0.84 ≤ α ≤ 0.94 for clinical and non-clinical samples (47–51).

### Procedure

First, the staff verbally informed the patients about the study. Then the patients received and signed the written information. Referring to the patients, the data took place in a separate, quiet room of the hospital at all three measurements time points. Controls were tested at the laboratories of the Clinical and Health Psychology Department in Ulm. Both groups were examined individually and tested three times based on the patients therapy process at the beginning (T1), after 4–6 weeks (T2), and at the end of therapy (T3).

The study started with the questionnaires prior to each testing session. Then the assessment of IA took place. At the end of each session, the staff assessed weight and size of the participants. Each session lasted about 45 min.

### Statistical Analysis

Group differences in age, BMI, IA, IS, and BDI-II were examined at T1 using *t*-tests. Change in the time course of therapy was assessed by repeated measures ANOVAs with *group* (AN group vs. control group) as between factor and *time* as within-subject factor in BMI, IA, IS, and BDI. Additional *post hoc* analyses were performed to examine differences between both groups for the different measurements. In addition, we reported η^2^ (eta-squared). Values less than 0.06 indicate small effects, between 0.06 and 0.14 mean medium effects, and higher than 0.14 announce large effects.

All statistical analyses were performed using Statistical Packages for Social Science (SPSS, version 21). A *p*-value less than 0.05 describes a significant result. If variances showed a difference in the Mauchly Test, the Greenhouse–Geisser correction values were used and uncorrected degrees of freedom were depicted.

## Results

### Sample Description and Questionnaire Data at T1

The relevant sample characteristics obtained from both participant groups are shown in Table 1. Results represent age, education, BMI, the performance in heartbeat perception (IA), scores in the subscale “interoceptive awareness” from the EDI-2 (IS), and BDI-II of the baseline measurement.

Anorectic patients had a significantly lower BMI, were less accurate in detecting their heartbeats (IA), reported more problems in IS as reflected in higher EDI scores, and scored higher in depression than healthy controls. No significant difference for educational level and age was observed. At the beginning of therapy, the difference between the two participant groups regarding BMI, IA, IS, and BDI-II was statistically significant.

### Change in BMI Over Time

**Figure 1:**
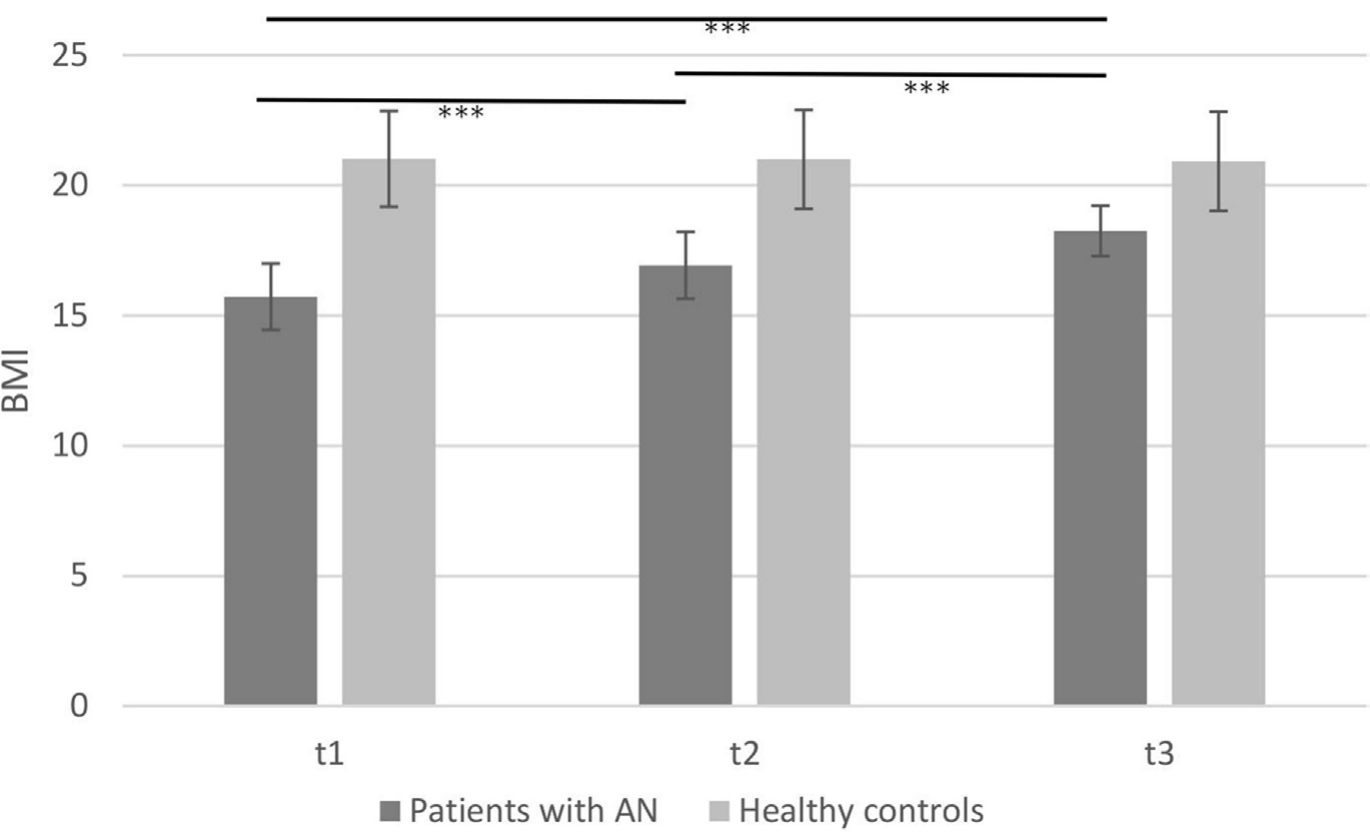
Mean body mass index of patients with anorexia nervosa compared to healthy controls in the time course of therapy (***p < 0.001; note: significant group differences present at all time points are not marked here).

The body weight among patients with AN recovered to almost normal range (see Figure 1) during an average of 12–14 weeks of therapy as assessed at three measurement points. At the beginning, the mean BMI of patients with AN was 15.72 (SD = 1.27) and increased to 18.25 (SD = 0.97) until the end of therapy. The analysis of variance on BMI revealed a main effect of *time course* [*F*(2, 56) = 81.02, *p* < 0.001; η^2^ = 0.74], a main effect of *group* [*F*(1, 28) = 50.73, *p* < 0.001; η^2^ = 0.64], and a *time* × *group* interaction [*F*(2, 56) = 93.35, *p* < 0.001; η^2^ = 0.77]. BMI of patients with AN increased over time, whereas BMI of healthy controls remained stable. *Post hoc* analyses among patients with AN showed significant differences in terms of BMI and the point of measurement (T1–T2: *p* < 0.001; T1–T3: *p* < 0.001; T2–T3: *p* < 0.001). As expected, the control group revealed no significant alteration for the BMI between the points of measurement (T1–T2: *p* = 1.00; T1–T3: *p* = 0.94; T2–T3: *p* = 1.00).

### Change in IA Over Time

**Figure 2:**
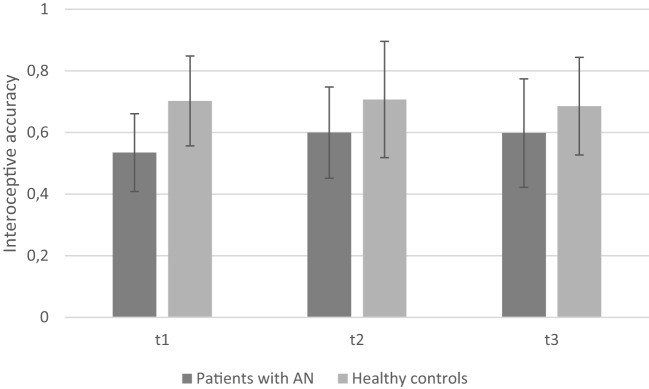
**Mean interoceptive accuracy of patients with anorexia nervosa compared to healthy controls depicting the three measurement points (note: significant group differences present at all time points are not marked here)**.

The mean IA scores of both groups (AN group and healthy controls) measured at three points in time are depicted in Figure 2.

The repeated measures ANOVA revealed a significant main effect of *group* [*F*(1, 28) = 5.99, *p* = 0.021; η^2^ = 0.18]: mean IA was significantly lower in AN at all three measurement points [mean T1 = 0.53 (SD = 0.13), mean T2 = 0.60 (SD = 0.15), and mean T3 = 0.60 (SD = 0.18)] as compared to healthy controls [mean T1 = 0.70 (SD = 0.15), mean T2 = 0.70 (SD = 0.19), and mean T3 = 0.69 (SD = 0.16)]. No significant main effect of *time course* [*F*(2, 56) = 0.92, *p* = 0.40; η^2^ = 0.03] and no *time* × *group* interaction [*F*(2, 56) = 1.29, *p* = 0.28; η^2^ = 0.04] were observed. Thus, there are no changes in IA over time for both groups.

### Change in IS (EDI-2) Over Time

**Figure 3:**
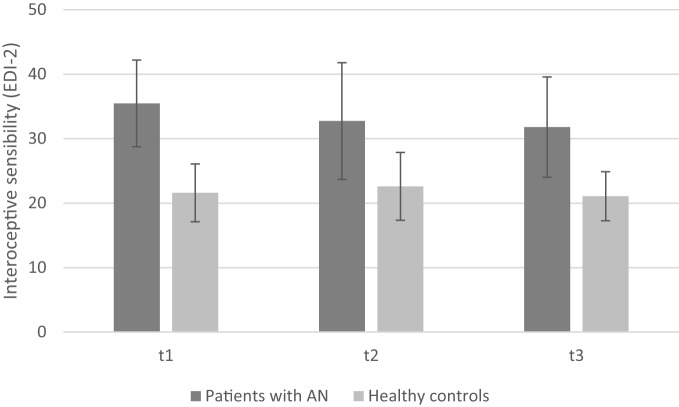
**Mean interoceptive sensibility of patients with anorexia nervosa compared to healthy controls depicting the three measurement points (note: significant group differences present at all time points are not marked here)**.

Interoceptive sensibility was measured by EDI-2 with higher scores indicating problems in IS. Results are depicted in Figure 3.

A significant main effect of *group* [*F*(1, 28) = 29.02, *p* < 0.001; η^2^ = 0.51] revealed more problems in IS in anorectic patients as compared to healthy controls. Furthermore, we observed a trend toward a main effect of *time course* [*F*(2, 56) = 3.17, *p* = 0.06; η^2^ = 0.10] and toward a *time* × *group interaction* [*F*(2, 56) = 2.86, *p* = 0.07; η^2^ = 0.09]. Problems in IS decreased in time course among patients with AN [mean T1 = 35.47 (SD = 6.72), mean T2 = 32.73 (SD = 9.06), and mean T3 = 31.80 (SD = 7.78)], whereas IS differed only slightly among healthy controls [mean T1 = 21.60 (SD = 4.48), mean T2 = 22.60 (SD = 5.26), and mean T3 = 21.07 (SD = 3.81); see Figure 3].

Exploratory *post hoc* analyses revealed that there are no significant differences for the AN group regarding the measurements from T1 to T2 (*p* = 0.31) and T2 to T3 (*p* = 0.97), but there is a marginal significant difference from T1 to T3 (*p* = 0.06). No significant differences were found among healthy controls (T1–T2: *p* = 1.00; T1–T3: *p* = 1.00; T2–T3: *p* = 0.44).

### Change in Depressive Symptoms (BDI-II) Over Time

**Figure 4:**
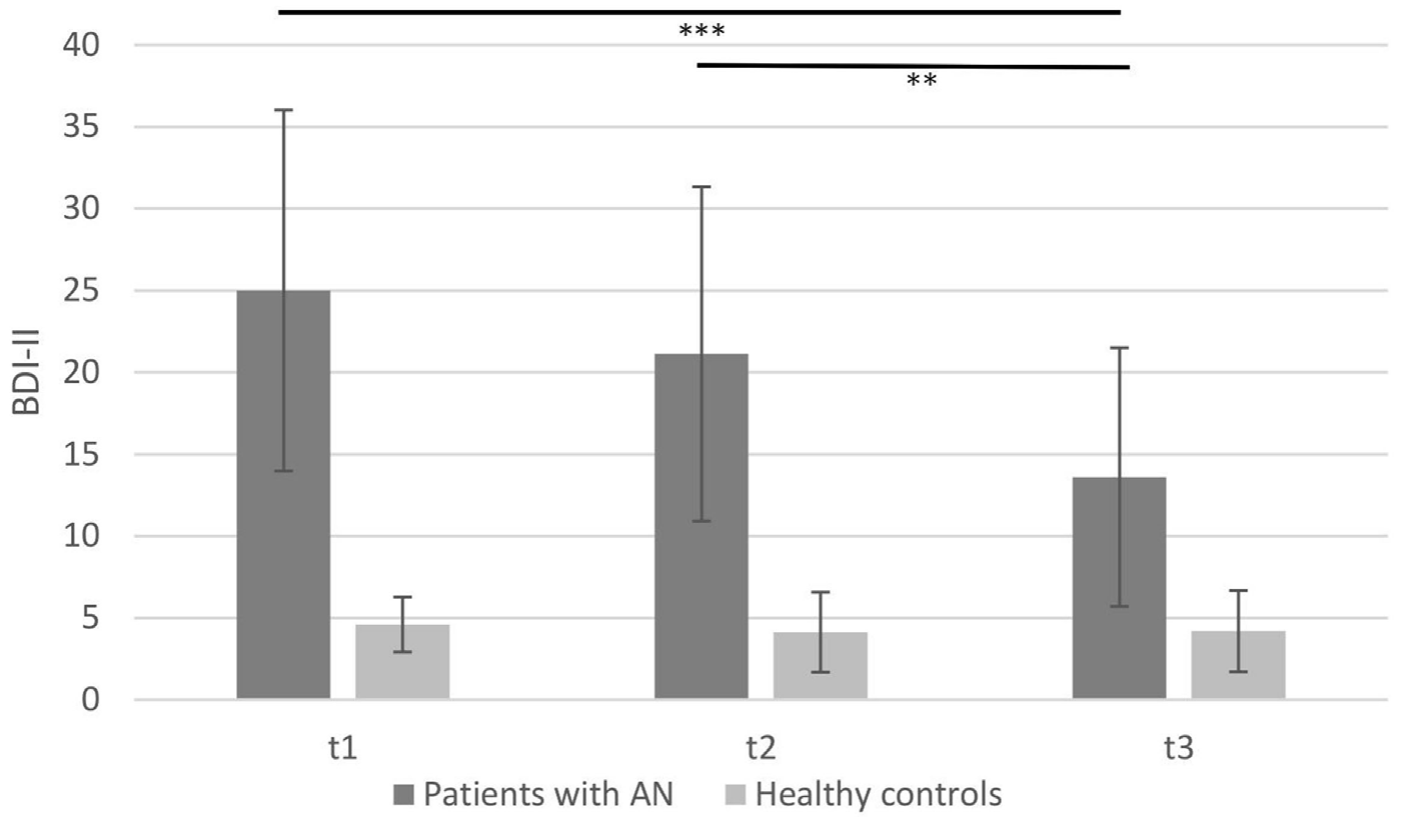
Mean Beck Depression Inventory-II of patients with anorexia nervosa compared to healthy controls depicting the three measurement points (**p < 0.01, ***p < 0.001; note: significant group differences present at all time points are not marked here).

Higher scores in BDI-II indicate depressive symptoms. Mean scores contrasting anorectic and controls in the time course are depicted in Figure 4.

There were significant main effects of *group* [*F*(1, 28) = 45.14, *p* < 0.001; η^2^ = 0.62], *time course* [*F*(2, 56) = 17.72, *p* < 0.001; η^2^ = 0.39], and a *time* × *group interaction* [*F*(2, 56) = 15.78, *p* < 0.001; η^2^ = 0.36]. Although the control group had a lower score during all three time points (see Figure 4), the AN group scored significantly higher on the BDI-II at time points T1, T2, and T3 [mean T1 = 25.00 (SD = 11.02), mean T2 = 21.13 (SD = 10.20), and mean T3 = 13.6 (SD = 7.90)]. This score decreased during therapy in the AN group. The *post hoc* testing showed no significant differences between T1 and T2 (*p* = 0.16). Nevertheless, there was a significant change from T1 to T3 (*p* < 0.001) and from T2 to T3 (*p* = 0.002) indicating that therapy induced an improvement of depressive symptoms for the AN group. Healthy controls showed no significant changes during the three measurements in the time course of 3 months [*F*(2, 28) = 0.37, *p* = 0.69].

## Discussion

This study investigated interoceptive processes in patients with AN in the time course of cognitive-behavioral therapy. Interoceptive processes were affected in anorectic patients, as measured in terms of IA and IS. Although we found some evidence for improvement in both IA and IS in anorectics in the time course of therapy, these effects were rather small and partly not consistent. Interpreting these results, it is important to notice that significant deficits in interoception remained in AN, while there were clear improvements referring to depressive symptoms or body weight.

Problems in self-reported IS were increased in anorectic patients, supporting earlier studies showing, e.g., a decreased ability to discriminate hunger and satiety in persons with different ED (6, 7, 29, 52). Moreover, Bruch (53) has postulated that a disturbed IS is a fundamental deficit among patients with AN. Following therapy, anorectic patients showed a trend toward an improvement in IS, especially when comparing T1 with T3. These findings are in accordance to previous studies suggesting that IS improved after therapy in AN. For example, Matsumoto et al. (6) found that anorectic patients had fewer problems in IS after therapy.

We observed a descriptive increase in IA as assessed by heartbeat perception from T1 to T2 in anorectic females, but this change did not reach significance, and there was no further improvement from T2 to T3. This result suggests that IA remains affected in AN in the time course of cognitive-behavioral therapy, probably creating a risk factor for ongoing disturbed processing of bodily signals. One can assume that observed deficits could be transferred to the perception of bodily signals in general, including sensitivity of bodily signals like hunger and satiety. This disturbed IA in patients with AN is in line with our findings showing a reduced IA for AN patients compared to the healthy controls (16).

To our knowledge, this is the first study examining possible changes in IA and related variables throughout the time course of therapy in patients with AN. One study that investigated IA in women recovered from bulimia nervosa in comparison to healthy controls also found an existing deficit in IA after therapy (54). Our study and the results from the study by Klabunde et al. (54) allow the conclusion that IA is a trait-like variable with ongoing disturbances in patients who suffered from AN that might act as a vulnerability factor for frequent relapses of patients with AN. Therefore, we need further research and more specific interventions to train and improve IA (e.g., biofeedback or bodily self-focused attention tasks).

First evidence indicates that IA can be improved in persons with rather low IA when they attend to their self as operationalized with looking in the mirror (26). Weisz et al. (55) used the same self-focused attention and showed partly an increase of IA. The presence of a mirror showed an effect on heartbeat discrimination, but not in terms of the heartbeat tracking performance. In further research, both methods should be critically discussed. Maybe it is easier for the participants to focus on the sound of the heartbeat in the heartbeat discrimination task, whereas at the heartbeat tracking task, participants’ beliefs about average heart rate might be reflected instead of focusing on the perception of their own heartbeat.

As we observed both deficits in IA and IS in AN, one can conclude that both aspects of interoceptive processes were affected in the same direction in this sample. Although changes in both subdomains were rather small, there was a trend toward an increase in IS in AN at T3, while the descriptively highest change in IA occurred between T1 and T2. These different time lines might suggest that the investigated both interoceptive domains might recover in a distinct order and might be related to different aspects of therapy. Whether other modalities such as gastrointestinal system that is closer related to eating pathology is also affected in the same extent like the investigated cardiovascular system is not yet clear, suggesting that other experimental measures like the waterload paradigm (21) might be useful in combination to the heartbeat detection task. A complex interaction between different modalities of internal systems was described by a recent study in panic disorder patients (56). Limmer et al. (56) reported an increased accuracy for cardiac signals alone, whereas IA for other internal signals like assessed skin conductance changes was perceived attenuated as compared to healthy controls.

Several strengths and limitations of this study should be noted. First, we examined a well-selected sample of patients with AN and without bulimia as well as matched healthy controls according to age and educational background. To our knowledge, this is the first study evaluating IA in time course of therapy. One limitation of this study is the small sample size due to the restrictive inclusion criteria obtained for study participants. Therefore, more data are needed to support these results. Furthermore, different comorbid symptoms like depression might interact with observed results, as some studies could demonstrate difficulties in IA for patients with depression (57) or a negative relationship between IA and depression in healthy participants (58). To sum up, our study established that IA did not improve or alter in patients with AN after successful therapy. Future studies should use longer follow-ups and additionally examine whether an increase in IA helps differentiating between relapsed and recovered patients.

## Author Contributions

SW, GB, MZ, TK, and OP designed the study. SW collected the data from the clinical group and DF collected the data from the control group. DF and OP conducted literature searches, provided summaries of previous research, and wrote first draft of the manuscript as well as conducted the statistical analyses. All the authors, DF, SW, GB, MZ, TK, and OP, wrote parts of the discussion section and contributed to and have approved the final manuscript.

## Conflict of Interest Statement

The authors declare that the research was conducted in the absence of any commercial or financial relationships that could be construed as a potential conflict of interest.
